# Adaptation and evaluation of the Nutrition Environment Measures Survey in Restaurants for the Spanish Mediterranean context (NEMS-R-MED)

**DOI:** 10.3389/fpubh.2025.1691374

**Published:** 2026-01-26

**Authors:** Eva María Trescastro-López, Alba Martínez-García, Esther Galilea Ramírez-Estrada, Sara Rey-Pérez, Lluís Català-Oltra, Cristóbal Llorens-Ivorra, María Tormo-Santamaría, Iván Hernández-Caravaca

**Affiliations:** 1 Department of Nursing, University of Alicante, Sant Vicent del Raspeig, Spain; 2 Balmis Research Group in History of Science, Health Care and Food, University of Alicante, Alicante, Spain; 3 Research Group on Applied Dietetics, Nutrition and Body Composition, University of Alicante, Alicante, Spain; 4 Alicante Institute for Health and Biomedical Research (ISABIAL, Group 23), Alicante, Spain; 5 Department of Community Nursing, Preventive Medicine and Public Health and History of Science, University of Alicante, Alicante, Spain; 6 University of Colima, Colima, Mexico; 7 University of Santiago de Compostela, Galicia, Spain; 8 Department of Sociology II, University of Alicante, Alicante, Spain; 9 Social Research Group on Equity and Diversity (EQUIDIVERSIDAD), University of Alicante, Alicante, Spain; 10 Public Health Center of Dénia (Alicante), Alicante, Spain

**Keywords:** food environment, measurement, NEMS-R-MED, restaurants, surveys and questionnaires, validation study

## Abstract

**Background:**

The Nutrition Environment Measures Survey in Restaurants (NEMS-R) is a validated tool originally developed in the United States for evaluating food environments in restaurants. This survey assesses the availability of healthy and unhealthy foods, the factors that facilitate or act as a barrier to healthy eating, food prices, and how foods are labeled and promoted. However, no such instrument exists for use in Spain, where the Mediterranean dietary context and restaurant culture differ significantly. The objective of this study was to adapt the NEMS-R instrument to the Spanish Mediterranean context (NEMS-R-MED) and evaluate its reliability and construct validity.

**Methods:**

Following a structured process of cultural adaptation—including translation, back-translation, expert review, and pilot testing—the NEMS-R-MED was applied independently by two raters in 57 restaurants across five neighborhoods in Alicante with varying socioeconomic statuses between November and December 2023. One of the raters evaluated the same restaurants again 30 days later. Inter-rater and intra-rater reliability were assessed using Cohen's kappa. Construct validity was tested using two techniques: (1) exploratory factor analysis and (2) known groups analysis, used to compare NEMS-R-MED scores between different types of restaurants and neighborhood socioeconomic levels.

**Results:**

Almost perfect inter-rater and intra-rater agreements were observed for most items (k > 80). Factor analysis revealed different constructs for each of the studied questions, which aligned with the theoretical constructs of the questionnaire. Significant differences in NEMS-R-MED scores were observed between sit-down restaurants, bar-cafeterias, and fast-food establishments, as well as across neighborhoods of different socioeconomic statuses, particularly in the “barriers to healthy eating” subscale.

**Conclusions:**

The adapted NEMS-R-MED instrument is a valid and reliable audit tool for assessing the food environment in restaurants within Spanish Mediterranean contexts. Additionally, NEMS-R-MED was able to discriminate effectively between restaurant types and neighborhoods with different socioeconomic statuses.

## 1 Introduction

Unhealthy diets have been linked to chronic non-communicable diseases (NCDs) and may be responsible for increased mortality rates worldwide (1, 2). Furthermore, both obesity and NCDs have doubled globally and in Spain in recent years (3–5). Dietary behaviors are complex and can be influenced by various factors (2), among which the food environment plays a decisive role, since it determines the accessibility, availability and promotion of food and can therefore shape dietary patterns and, consequently, population health (6, 7). In this regard, mass catering plays a fundamental role, since restaurant environments are characterized by the extensive advertising of unhealthy foods that are rich in sugars, fats, salt and calories and by eating affordable, together with a food supply that is scarce in vegetables such as legumes and fruit (8). According to the 2023 Report on Food Consumption in Spain, spending on eating outside the home increased by 5.8% last year (9). Furthermore, expenditure on eating out exceeded 34,900 million euros in 2023, accounting for around 30% of total food costs (10). Likewise, some research suggests that frequent use of mass catering for leisure, tourism or work purposes may be associated with weight gain and the development of overweight and obesity (11–13).

Therefore, a tool that can evaluate the food environment in restaurants is necessary. Several instruments are currently available to measure food environments (14–16). Of the existing instruments, those developed by Glanz et al. are among the most widely used to characterize the food environment (11, 14–17). Known as the Nutrition Environment Measures Survey (NEMS), they were developed in the United States for an American context. Specifically, the versions that have been most widely adapted and used for developing other instruments internationally are those aimed at identifying the food environment in stores and restaurants (17). The Nutrition Environment Measures Survey in Restaurants (NEMS-R) assesses restaurant food environments by focusing on different types of food indicators: healthy/unhealthy dish options and beverages, children's menus, signage and promotions of food, facilitators and barriers to healthy eating, pricing, and accessibility (11). In this regard, facilitators of healthy eating in restaurants include the availability of healthy dishes, clear nutritional information, the option to customize dishes, adequate portion sizes or affordable prices. Conversely, barriers are factors that make healthy eating more difficult, such as limited healthy options, excessive portion sizes, a lack of ingredient information, the promotion of high-fat, high-sugar or high-salt foods, or the high prices of healthier dishes (11). These conditions may influence customers' decisions and their ability to maintain adequate eating habits when eating out.

As the Spanish Mediterranean context and food culture differ from those in which the instrument was validated, it is essential to adapt the tool in order to determine the characteristics of the food environments in restaurants in the Spanish context. Additionally, in Spain, only two instruments of this type have been adapted and validated to date: the Nutrition Environment Measures Survey in Food stores Spanish Mediterranean Context (NEMS-S-MED), which assesses the availability and affordability of healthy foods in food stores (18), and the Perceived Nutrition Environment Measures Survey to the Mediterranean Spanish Context (NEMS-P-MED), which measures the individuals' perception of different types of food environment (food stores, restaurants and the home) (19). However, no tools currently exist to assess the food environment in restaurants.

For all the aforementioned reasons, this study aims to adapt and evaluate the NEMS-R instrument for the Spanish Mediterranean context (NEMS-R-MED). This will provide a valid and reliable tool for assessing the availability, facilitators and barriers to healthy foods in the restaurant environment in Spain.

## 2 Materials and methods

### 2.1 Study design and sample

The NEMS-R survey was culturally adapted and validated for the Spanish context, in line with the process outlined by Ramada-Rodilla et al. (20). This process included two stages: (a) the transcultural adaptation of the instrument, consisting of the translation, back-translation, expert committee review and pilot testing of the scale; and (b) the validation of the instrument, comprising the verification of the properties determining intra- and inter-rater reliability and construct validity.

### 2.2 Development of the NEMS-R-MED

#### 2.2.1 Translation and transcultural adaptation

After obtaining consent from the research group that developed the NEMS-R survey (11), the translation and transcultural adaptation processes were carried out. This involved translating and interpreting the original English-language questionnaire into Spanish. To achieve this, two translations were produced independently by two experts: one was a native Spanish speaker and the other was an English interpreter specializing in health sciences.

Next, an expert committee comprising a multidisciplinary team made up of dietitians and nurses assessed the two translations grammatically, linguistically and semantically. The aim was to reach an agreement on the final questions, taking into account the quality of the adaptation of the expressions and the relevance, clarity and cultural appropriateness of the items in the survey for the Spanish context.

#### 2.2.2 Pilot testing

A pilot test was then conducted using the preliminary version of the questionnaire. These data were excluded from the main study. The objective of the pilot test was to assess the correctness of the translation, determine the feasibility of the survey and evaluate its overall comprehensibility. The pilot test enabled adjustments to be made to the questionnaire, including the elimination or modification of items, thereby defining the NEMS-R-MED survey. The suffix “MED” was added due to its application in the Mediterranean context and because there are already NEMS questionnaires adapted to the Spanish context with this designation (18, 19).

#### 2.2.3 Modifications and final survey

The NEMS-R-MED instrument was developed by adapting the items in the NEMS-R version to the Spanish population (21, 22).

The original survey comprises 25 questions assessing aspects related to the restaurant, as well as the availability of items in multiple menu categories, including healthy main dish choices, the availability of fruits and vegetables without added sauce, wholegrain bread and baked chips, signage and promotions, facilitators and barriers to healthy eating, pricing and children's menus (11). Supplementary Table S1 provides further information on the questions related to food availability.

In the Spanish version, food and beverage items were organized into the following categories: Specific Food Availability, Starters, Main Courses, Desserts and Beverages. Additionally, the NEMS-R-MED included specific variables characteristic of the Mediterranean diet, such as legumes, fish, rice dishes, stews and soups, which were included in both the starters and main courses. Sweets and dairy products were also included in the desserts, and alcohol and water in the beverages category. Furthermore, given the characteristics of the Spanish context, where it is common for daily set menus in restaurants not to be shared, the following item was added in the questionnaire: “The obligation to order one menu per diner.”

At the beginning of the survey, information about the restaurant is requested, including its type (sit-down restaurant, bar-cafeteria, or fast-food), name, opening hours, and accessibility features (adapted access, parking, room capacity, number of tables).

The adapted NEMS-S-MED instrument finally included 16 questions and 63 items assessing food options (starters, main courses and desserts) and available drinks (34 items), as well as the facilitators or barriers to healthy eating (four and five items, respectively), price comparisons (five items) and the presence of a children's menu (15 items assessing the availability of food on the children's menu). Table 1 displays the categories, the number of questions in each category, and the items included. The questions employed different types of response formats: dichotomous (yes/no), ordinal with a Likert-type scale of options for questions related to price [More, Same, Less, Not applicable (N/A)], and direct notation (for example: number of options).

**Table 1:** Categories and items included in the adapted Nutrition Environment Measures Survey in Restaurants for Mediterranean contexts (NEMS-R-MED) audit tool.

**Categories**	**Number of questions**	**Items included**
Specific foods availability	6	Chips
		Baked chips
		White bread
		100% wheat or whole grain bread
		Healthy salads
		Salads with unhealthy sauces
Starters	7	Healthy choices
		Salads as a standalone dish
		Unfried vegetables
		Grilled/boiled/baked fish
		Meat
		Legumes
		Fried or battered products
Main dishes	9	Healthy main dish options
		Salads as a standalone dish
		Unfried vegetables
		Grilled fish
		Meat
		Legumes
		Rice
		Pasta
		Stews and soups
Desserts	4	Sweets (cakes, pies, brownies,…)
		Sweetened dairy products (ice cream, yogurt, custard,…)
		Unsweetened dairy products
		Fruit
Beverages	8	100% fruit juice
		Juice drink
		Sweetened soft drinks
		Light/zero soft drinks
		Alcohol (wine/beer)
		Water
		Possibility to order water free of charge
		Other healthy unsweetened beverages (coffee, tea,…)
Facilitators of healthy eating	4	Nutrition information
		Healthy entrees identified on the menu
		Reduced portion size
		Comments encouraging healthy eating
Barriers to healthy eating	5	Restaurant menu or signage promotes unhealthy choices
		Menu or restaurant signage encourage overeating
		Large portion encouraged
		Possibility of increasing the size of the portions
		Comments that discourage ingredient changes
Pricing	5	Sum of individual dishes vs. combined menu
		Healthy starters vs. unhealthy starters
		Obligation to order one menu per diner
		Reduced or half portions vs. regular portions
		Soft drinks vs. water
Kid's menu	15	Healthy options
		100% fruit juice
		Juice drink
		Water
		Sweetened soft drinks
		Light/zero soft drinks
		Refills of unhealthy drinks.
		Healthy garnish available.
		Replacing a healthy option with an unhealthy one (e.g., salad for French fries)
		Dishes with assigned healthy garnish (e.g., vegetables or salads)
		Unhealthy dessert (e.g., sweets, cakes, pies, ice cream, etc.)
		Healthy dessert (e.g., fruit)
		Nutrition information
		Promotion of unhealthy foods
		Promotion of healthy foods

The original survey score ranges from −5 to 21 in the total range without a children's menu, and from −8 to 30 in the total range complete with a children's menu (11). The final NEMS-R-MED score was also adjusted to range from −5 to 23 when no children's menu is available, and from −10 to 31 when children's menu is included (Table 2). This difference in points is due to the inclusion of certain foods (such as legumes or unsweetened dairy products) in the adapted score. This means that the higher the score, the greater the range and affordability of healthy options offered by the restaurant. The scores can be used independently–with or without a children's menu- without affecting their validity.

**Table 2:** NEMS-R-MED scoring.

**Categories**	**Variable**	**Score**
Availability of food	Baked chips	Yes = 1 pt
	100% wheat or whole grain bread	Yes = 1 pt
	100% fruit juice	Yes = 1 pt
	Water and other unsweetened beverages (tea, herbal teas, coffee, etc.)	Yes to either one = 1 pt
	Healthy entrees	1 option = 1 pt
		2–4 options = 2 pt
		+5 options = 3 pt
	Salads as a standalone dish (both as starters and main courses)	1 option = 1 pt
		2–4 options = 2 pt
		+5 options = 3 pt
	Healthy salads	1 option = 1 pt
		2–4 options = 2 pt
		+5 options = 3 pt
	Legumes (both in starters and main dishes)	Yes = 1 pt
	Unfried vegetables	Yes = 1 pt
	Unsweetened dairy products	Yes = 1 pt
	Fruit	Yes = 1 pt
	Range	0 a 17
Facilitators of healthy eating	Healthy options identified in both starters and main courses	Yes = 1 pt
	Nutrition information	Yes = 1 pt
	Healthy entrees	Yes = 1 pt
	Reduced portion size	Yes = 1 pt
	Comments encouraging healthy eating	Yes = 1 pt
	Healthy starters cheaper than normal starters	Yes = 1 pt
	Range	0 a 6
Barriers to healthy eating	Restaurant menu or signage promotes unhealthy choices	Yes = −1 pt
	Menu or restaurant signage encourages overeating	Yes = −1 pt
	Large portions encouraged	Yes = −1 pt
	Possibility of increasing the size of the portions	Yes = −1 pt
	Comments that discourage ingredient changes	Yes = −1 pt
	Range	−5 a 0
Kid's menu	Healthy options	Yes = 1 pt
	100% fruit juice	Yes = 1 pt
	Water	Yes = 1 pt
	Sugary drinks (soft drinks and sugary juices)	Yes to either one = −1 pt
	Refills of unhealthy drinks.	Yes = −1 pt
	Healthy garnish available.	Yes = 1 pt
	Replacing a healthy option with an unhealthy one (e.g., salad for French fries)	Yes = −1 pt
	Dishes with assigned healthy garnish (e.g., vegetables or salads)	Yes = 1 pt
	Unhealthy dessert (e.g., sweets, cakes, pies, ice cream, etc.)	Yes = −1 pt
	Healthy (e.g., fruit)	Yes = 1 pt
	Nutrition information	Yes = 1 pt
	Promotion of unhealthy foods	Yes = −1 pt
	Promotion of healthy foods	Yes = 1 pt
	Range	−5 a 8

The survey and scoring of NEMS-R-MED are found in the Supplementary material.

### 2.3 Data collection

#### 2.3.1 Data collection process

##### 2.3.1.1 Training of raters

First, the personnel were trained. The two raters were dietitians who had undergone training to conduct the survey over a 2-week period. During the first week, two initial face-to-face sessions were held at the university, led by the project's principal investigator. In these sessions, the survey questions were reviewed and discussed, and the raters were instructed on how to collect the data in each scenario. Subsequently, the raters spent a week testing the audit tool in local restaurants to familiarize themselves with it. A final session was held to answer any remaining questions and confirm that the raters were ready to collect the data.

##### 2.3.1.2 Neighborhoods selection

**Figure 1:**
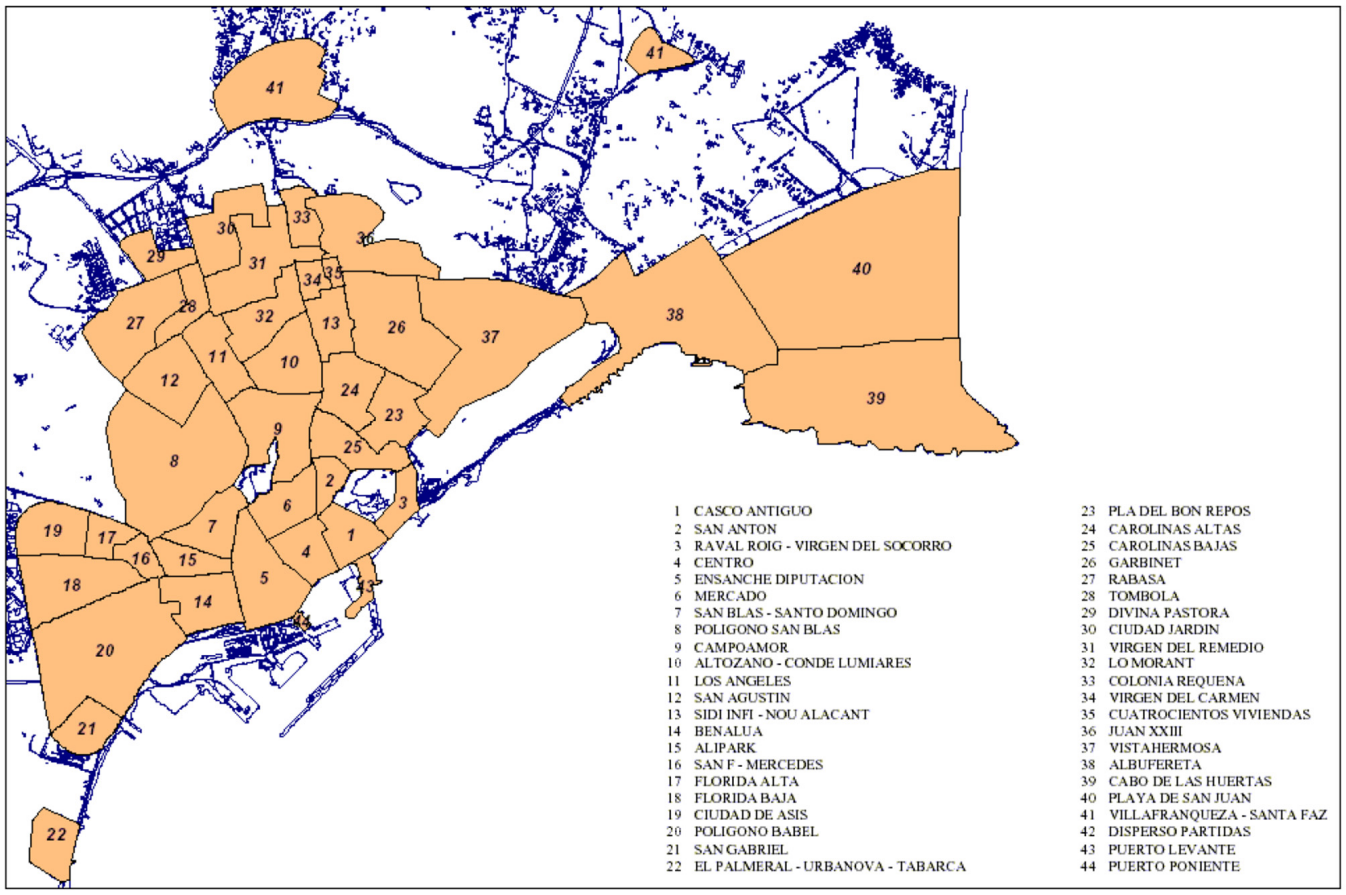
Neighborhoods of Alicante. Reproduced from “Alicante neighborhood (Ask Hinzel)” by Kokoo, licensed under CC BY-SA 3.0.

Prior to data collection, neighborhoods were selected based on predefined socioeconomic criteria. To identify the neighborhoods in Alicante, the Urban Audit programme was used, which compiles data from the National Institute of Statistics of Spain from 2020 (23), as this was the most recent dataset available. In order to select the neighborhood that best represented the population, data on income, dependency rate, the proportion of foreign residents and population weight were considered. The following neighborhoods, which have different socioeconomic statuses (SES), were finally included: (1) “*Colonia Requena*” [low SES; map code (MP) 33; see Figure 1, Neighborhoods of Alicante], (2) “*Virgen del Carmen*” (low SES; MP 34), (3) “*Mercado*” (medium SES; MP 6), (4) “*Campoamor*” (medium SES; MP 9) and (5) “*Cabo de las Huertas*” (high SES; MP 39).

##### 2.3.1.3 Fieldwork and data collection period

For data collection, they followed a predefined route within each neighborhood included in the study to guide the fieldwork. A specific route was designed for each neighborhood to ensure that all streets were covered. Beginning with the neighborhoods in the north of the city, the route was then extended to the remaining areas. Between November and December 2023, all surveys were completed on weekdays.

##### 2.3.1.4 Inclusion and exclusion criteria

During this stage, inclusion and exclusion criteria for restaurants were applied. These criteria are detailed below. Inclusion criteria: all restaurants located in the selected neighborhoods were eligible for data collection. Exclusion criteria: establishments within the selected neighborhoods that were not open to the general public (schools, hospitals, soup kitchens, cafeterias and canteens for minors under 18, workplace cafeterias), those that charge an entrance fee (museum cafeterias, monuments, theaters, etc.), those that do not have a menu, and those that are not open all year round.

#### 2.3.2 Inter-rater and intra-rater reliability

To assess inter-rater reliability (i.e., the degree to which measures are consistent between two or more evaluators) (20), the restaurants were visited and surveyed independently on the same day by two data collectors. To assess intra-rater reliability (i.e., the consistency of a measurement when replicated over time) (20), one of the original raters re-evaluated all of the restaurants ~30 days after the initial observations.

#### 2.3.3 Construct validity

To assess construct validity (i.e., the extent to which the tool's measurements align with theoretical hypotheses) (20), the data collected by rater 1 during the initial data collection were utilized. Similar to previous studies (11, 18), known groups validity was analyzed to determine whether differences existed according to socioeconomic status and restaurant type.

### 2.4 Statistical analysis

#### 2.4.1 Inter-rater and intra-rater reliability

Inter-rater and intra-rater reliability were assessed using percent agreement and kappa coefficients (κ) for all binary and categorical items. According to the guidelines of Landis and Koch, the following cut-off ranges for kappa values were used: < 0 (poor), 0.0–0.20 (slight), 0.21–0.40 (fair), 0.41–0.60 (moderate), 0.61–0.80 (substantial), and 0.81–1.00 (almost perfect) (24).

#### 2.4.2 Construct validity

Construct validity was assessed using two different techniques: (1) exploratory factor analysis and (2) known groups analysis. First, the exploratory factor analysis was conducted to identify how many factors made up each item related to the food environment in restaurants. In this regard, the survey included items that measured various theoretical constructs, which were identified through factor analysis. Some questions distinguished between the availability of healthy and unhealthy food and drink, while others differentiated between factors that facilitate or hinder healthy choices relating to menu information, marketing, price or portion size. To confirm whether factor analyses could be conducted with the survey items, the Kaiser-Meyer-Olkin (KMO) test was performed along with Bartlett's Test of Sphericity. For factor analysis to be appropriate, the KMO value needed to be >0.5, and the *p*-value of Bartlett's sphericity test had to be significant (*p* < 0.05) (25).

Secondly, to ascertain whether the tool's measurements aligned with the theoretical hypotheses, a known groups analysis was performed. To determine whether the NEMS-R-MED score could discriminate between restaurants of different types and areas of different socioeconomic statuses, the scores were compared. Based on previous research (11), it was hypothesized that sit-down restaurants would offer a greater variety of healthy food options than fast-food restaurants. The second hypothesis was that restaurant scores would be lower in neighborhoods with a lower socioeconomic status. The tool was considered valid if the hypothesis were congruent with the expected results.

As only 10 of the 57 restaurants included had children's menus, it was decided to analyse the data using the survey score that did not take them into account, in order to ensure comparability across scores. Comparisons between restaurant types and SES were made using the Chi-square test for dichotomous variables and *t*-test or the Kruskal–Wallis test for continuous variables (depending on the variable distribution). Statistical significance was set at *p* < 0.05. All data management and analyses were conducted using IBM SPSS Statistics v28.0.0.

## 3 Results

### 3.1 Descriptive data

A total of 57 restaurants were identified across the selected neighborhoods in Alicante. Of these, 18 were sit-down restaurants, 33 were bar-cafeterias and six were fast-food establishments. A smaller number of sit-down restaurants was observed in neighborhoods with a lower socioeconomic status (*n* = 6). The distribution of restaurants types by neighborhood and socioeconomic status is presented in Table 3.

**Table 3:** Percentage of restaurant types in each neighborhood, by socioeconomic status (SES).

**Type of restaurant**	**Total**		**High SES neighborhoods**		**Medium SES neighborhoods**		**Low SES neighborhoods**	
	***n***	**%**	***n***	**%**	***n***	**%**	***n***	**%**
Sit-down restaurant	18	31.6	8	14.0	9	15.8	1	1.8
Bar-cafeteria	33	57.9	10	17.5	18	31.6	5	8.8
Fast food	6	10.5	2	3.5	4	7.0	0	0.0
Total	57	100.0	20	35.0	31	54.4	6	10.6

### 3.2 Inter-rater and intra-rater reliability

Both inter-rater and intra-rater reliability were consistently very high. As shown in Table 4, 42 of the 50 items showed almost perfect inter-rater agreement (κ > 0.80), and 39 items demonstrated the same level of intra-rater agreement. Substantial agreement (κ = 0.61–0.80) was observed for seven and nine items, respectively, while moderate agreement applied to two items in both analyses. Fair agreement was only found for intra-rater reliability for the item “Comments that discourage ingredient changes” (κ = 0.345).

**Table 4:** Reliability of the Nutrition Environment Measures Survey in Restaurants for Mediterranean contexts (NEMS-R-MED).

**Categories**	**Item**	**Inter-rater reliability (*****n*** **=** **57)**		**Intra-rater reliability (*****n*** **=** **57)**	
		**Percent agreement**	**Cohen Kappa**	**Percent agreement**	**Cohen Kappa**
Specific foods availability	Chips	100.0	1.000	100.0	1.000
	Baked chips	100.0	1.000	100.0	1.000
	White bread	95.6	0.782	100.0	1.000
	100% wheat or whole grain bread	100.0	1.000	98.3	0.965
	Healthy salads	91.2	0.795	91.2	0.795
	Salads with unhealthy sauces	80.7	0.624	91.2	0.824
Starters	Healthy choices	100.0	1.000	94.8	0.876
	Salads as a standalone dish	100.0	1.000	96.5	0.855
	Unfried vegetables	94.7	0.698	94.7	0.698
	Grilled/boiled/baked fish	96.5	0.648	96.5	0.486
	Meat	96.5	0.919	94.7	0.872
	Legumes	96.5	0.814	93.0	0.563
	Fried or battered products	100.0	1.000	93.0	0.856
Main dishes	Healthy main dish options	91.2	0.744	94.8	0.854
	Salads as a standalone dish	100.0	1.000	94.7	0.890
	Unfried vegetables	94.7	0.811	91.3	0.616
	Grilled fish	98.3	0.957	91.3	0.787
	Meat	100.0	1.000	98.3	0.848
	Legumes	96.5	0.895	98.2	0.789
	Rice	98.2	0.961	96.5	0.923
	Pasta	96.5	0.905	94.1	0.854
	Stews and soups	100.0	1.000	96.5	0.894
Desserts	Sweets (cakes, pies, brownies,…)	100.0	1.000	94.8	0.854
	Sweetened dairy products (ice cream, yogurt, custard,…)	91.2	0.821	89.4	0.784
	Unsweetened dairy products	100.0	1.000	98.3	0.659
	Fruit	100.0	1.000	94.7	0.847
Beverages	100% fruit juice	96.5	0.923	94.7	0.886
	Juice drink	98.2	0.931	100.0	1.000
	Sweetened soft drinks	100.0	1.000	100.0	1.000
	Light/zero soft drinks	98.3	0.848	98.2	0.791
	Alcohol (wine/beer)	100.0	1.000	100.0	1.000
	Water	100.0	1.000	100.0	1.000
	Possibility to order water free of charge	98.2	0.913	95.0	0.824
	Other healthy unsweetened beverages (coffee, tea,…)	96.4	0.868	98.2	0.931
Facilitators of healthy eating	Nutrition information	100.0	1.000	100.0	1.000
	Healthy entrees identified on the menu	86.0	0.513	93.0	0.632
	Reduced portion side	100.0	1.000	100.0	1.000
	Comments encouraging healthy eating	100.0	1.000	100.0	1.000
Barriers to healthy eating	Restaurant menu or signage promotes unhealthy choices	96.4	0.887	96.5	0.879
	Menu or restaurant signage encourage overeating	96.5	0.814	98.2	0.913
	Large portion encouraged	94.7	0.701	100.0	1.000
	Possibility of increasing the size of the portions	94.7	0.872	97.2	0.959
	Comments that discourage ingredient changes	87.7	0.593	85.2	0.345
Pricing	Sum of individual dishes vs. combined menu	98.2	0.958	98.3	0.956
	Healthy starters vs. unhealthy starters	86.0	0.715	86.0	0.702
	Obligation to order one menu per diner	94.7	0.856	77.2	0.540
	Reduced or half portions vs. regular portions	93.0	0.893	91.2	0.863
	Soft drinks vs. water	100.0	^a^	100.0	^a^
Kids' menu	Availability	100.0	1.000	95.1	0.810
	Healthy options available	96.5	0.880	93.0	0.721

^a^A Kappa could not be calculated because the variable was constant.

The highest inter-rater agreement (κ = 1.00) was observed for chips, baked chips, 100% whole-wheat or wholegrain bread, healthy choices, salad as a standalone dish, fried or battered products, meat, stews and soups, sweets (cakes, pies, brownies, etc.), unsweetened dairy products, fruit, alcohol, water, nutrition information, reduced portion sizes and comments encouraging healthy eating. The highest intra-rater agreement was found for chips, baked chips, white bread, alcohol, water, nutrition information, reduced portion sizes and comments encouraging healthy eating. The items with the lowest inter- and intra-rater reliability scores were “Healthy entrees identified on the menu” and “Comments that discourage changes to ingredients.”

### 3.3 Construct validity

The construct validity was conducted independently for each question in the factor analysis, as each of them measured different constructs of the food environment. The KMO and Barlett tests gave satisfactory results [KMO >0.5; Barlett (*p* ≤ 0.05)] in the following multiple-item questions: 6a, 6b, 7a,7b (KMO = 0.563; Barlett X2 = 27.509, *p* < 0.001), 12a, 12b, 12c, 12d, 12e, 12g, 12h (KMO = 0.538; Barlett X2 = 126.015, *p* < 0.001), 13a, 13b, 13c, 13d (KMO = 0.595; Barlett X2 = 51.527, *p* < 0.001), and 14a, 14b, 14c, 14d, 14e (KMO = 0.670; Barlett X2 = 83.514, *p* < 0.001).

Table 5 shows the results of the factor analysis. Two constructs were identified in the first analyzed question (Specific foods availability): (1) the presence of healthy foods, and (2) the presence of unhealthy foods. The subsequent question regarding beverages revealed three factors: (1) The availability of healthy beverages; (2) The availability of sugary/unhealthy beverages; and (3) The availability of alcoholic drinks. The following two questions, concerning facilitators and barriers to healthy eating in restaurants, highlight two factors. In this case, the items were divided into two categories: those related to menu information (whether healthy or not), and those addressing issues associated with increasing, reducing or modifying portion sizes.

**Table 5:** Exploratory factor analysis by factors according to the multi-item questions contained in the NEMS-R-MED survey.

**Item**	**Specific foods availability**		**Beverages**			**Facilitators of healthy eating**		**Barriers to healthy eating**	
	**F1**	**F2**	**F1**	**F2**	**F3**	**F1**	**F2**	**F1**	**F2**
6a	0.894								
6b		0.783							
7a	0.865								
7b		0.754							
12a				0.850					
12b			0.760						
12c			0.829						
12d			0.901						
12e					0.854				
12g				0.790					
12h				0.671					
13a						0.834			
13b						0.719			
13c							0.971		
13d						0.894			
14a								0.809	
14b								0.890	
14c								0.877	
14d									0.971
14e									0.616

Extraction method: analysis of main components. Rotation method: varimax with Kaiser standardization. F, factor.

Tables 6, 7 present the results of the known groups analysis, confirming the proposed hypotheses. The scores by type of establishment show that sit-down restaurants received the highest scores overall and across all subcategories, followed by bars/cafés and finally fast-food outlets (Table 6). A statistically significant association was observed between the overall score and the facilitators subcategory (*p* < 0.05). Table 7 shows that the total score was higher in neighborhoods with the highest socioeconomic status, followed by those with medium status, and finally those with a low status. No statistically significant differences were observed for the total score, although differences were found in the barrier's subscale.

**Table 6:** NEMS-R-MED scores by restaurant type.

**Scoring**	**Sit-down restaurant** ***N*** **= 18**			**Bar-cafeteria** ***N*** **= 33**			**Fast food outlets** ***N*** **= 6**			**Sig.^a^**
	**Mean**	**SD**	**[min, max]**	**Mean**	**SD**	**[min, max]**	**Mean**	**SD**	**[min, max]**	
Availability of healthy foods	5.67	2.57	[2, 11]	4.85	2.21	[1, 12]	3.83	2.71	[1, 9]	0.135
Facilitators of healthy food	2.11	1.02	[1, 5]	1.55	0.79	[0, 3]	0.83	0.31	[0, 2]	0.012
Barriers to healthy food	−1.11	0.68	[−2, 0]	−1.27	0.98	[−4, 0]	−2.17	0.60	[−4, −1]	0.282
Total	6.67	2.78	[2, 12]	5.12	2.80	[−2, 13]	2.5	3.99	[0, 10]	0.019

^a^Sig. correspond to Kruskal–Wallis test.

**Table 7:** NEMS-R-MED scores by socioeconomic status (SES) of the neighborhood.

**Scoring**	**High SES neighborhoods** ***N*** **= 20**			**Medium SES neighborhoods** ***N*** **= 31**			**Low SES neighborhoods** ***N*** **= 6**			**Sig.^a^**
	**Mean**	**SD**	**[min, max]**	**Mean**	**SD**	**[min, max]**	**Mean**	**SD**	**[min, max]**	
Availability of healthy food	5.25	0.45	[1, 9]	4.93	2.76	[2, 12]	4.50	1.52	[3, 7]	0.393
Facilitators of healthy food	1.85	1.04	[0, 5]	1.45	0.85	[0, 4]	2.00	0.89	[1, 3]	0.220
Barriers to healthy food	−1.10	0.64	[−2, 0]	−1.29	1.10	[−4, 0]	−2.17	0.40	[−4, −1]	0.040
Total	6.00	2.41	[0, 9]	5.09	3.56	[−2, 13]	4.33	2.73	[0, 8]	0.138

^a^Sig. correspond to Kruskal–Wallis test.

## 4 Discussion

This study adapted and evaluated the NEMS-R-MED survey for the Spanish context. The findings indicate that the instrument is valid and reliable for assessing availability, facilitators and barriers of healthy foods in the food environment of restaurants.

Intra-rater and inter-rater reliability data were almost perfect for most items, aligning with the original validation (11) and subsequent adaptations in other contexts (17, 26) and countries such as Australia (27), Brazil (28) or China (29). The original NEMS-R has been widely used and adapted in different contexts such as military, university, hospital, or school cafeterias (17), obtaining robust and relevant data.

In this study, some values decreased in terms of intra-rater reliability, such as grilled, boiled or baked fish and legumes in starters and main dishes, and unfried vegetables in main dishes, as well as unsweetened dairy products in desserts. This may have been because the data were collected around 30 days later. During this time, the food available at the surveyed restaurants may have varied due to changes in the menu, as this is characteristic of the Spanish Mediterranean context (30).

With regard to construct validity, the factorial analysis revealed different constructs for each of the studied questions. As discussed in the Results section, the question regarding the availability of specific foods distinguishes between two constructs: healthy and unhealthy foods. In the case of beverages, three factors are covered: healthy beverages; sugary/unhealthy beverages; and alcoholic beverages. Regarding the questions about facilitators and barriers to healthy eating in restaurants, two categories stand out: those referring to menu information (whether healthy or not), and those addressing issues related to increasing, reducing, or modifying portion sizes. These factors are consistent with the theoretical constructs of the questionnaire, demonstrating construct validity. Although no factor analysis was performed in the original survey, it was determined that the tool was useful due to the numerous significant differences in food environment variables across restaurant types. This can be interpreted as supporting the construct validity of the variables (11).

Furthermore, NEMS-R-MED was able to discriminate between restaurant types and neighborhoods with different socioeconomic statuses. Similar to previous research (11, 31–33), fast-food restaurants obtained lower scores than sit-down restaurants. Previous studies indicate that the healthiness of food environments worldwide varies by neighborhood, with low-income areas having a higher concentration of supermarkets, takeaways and fast food outlets (34–36). Furthermore, evidence suggests that inequalities in access to food depend on socioeconomic characteristics of the habitat (35, 37). In this sense, the results showed that the wealthy neighborhood scored higher. However, no statistically significant associations were observed for the total score. Nevertheless, a statistically significant relationship was found for the “Barriers” sub-score. It was observed that there were more barriers to healthy eating in poorer neighborhoods and that this trend appeared gradually across neighborhoods. This is consistent with data from previous studies. For instance, a study conducted in Brazil observed that healthy foods in restaurants were more readily available in neighborhoods with a higher socioeconomic status (28). Another study conducted in Australia observed differences in NEMS-R scores across neighborhoods. It found a significantly more supportive restaurant food environment in the high-SES neighborhood, with greater access to and availability of healthy foods, as well as reduced barriers to healthy eating and substantially more nutrition information (38).

In Spain, little evidence exists on the food environment in restaurants. However, it has been observed that stores in neighborhoods with a higher socioeconomic status tend to have a greater availability of healthy foods. This has been found in stores in different cities such as Madrid (39), Barcelona (40) and Alicante (41). Moreover, when comparing the results of the Alicante study with the present one, it should be noted that, as with the number of restaurants, which is lower in the most disadvantaged neighborhoods, there are also fewer stores in those neighborhoods with fewer resources, making access to food even more difficult in this city. These data support the findings from several previous worldwide studies, which indicate that people with a lower socioeconomic status are relatively more exposed to unhealthy food environments (42–44). This situation, combined with the fact that a lack of economic resources can reduce the affordability of healthy foods, highlights how eating behavior is influenced by the food environment.

This study has the following limitations. In order to obtain more conclusive data on the relationship between neighborhood socioeconomic status, it would be necessary to increase the sample size. Despite this, the current sample is adequate for validating the instrument. Moreover, further studies in other areas are needed to assess how generalisable the measure and findings are.

Notwithstanding these limitations, this study also boasts significant strengths. To the best of our knowledge, this is the first study to adapt and assess the validity of the NEMS-R tool in Spain. This will provide valid and reliable tools for characterizing the food environment in restaurants in the Spanish Mediterranean context.

Finally, it should be noted that the adaptation and validation of the NEMS-R-MED (Nutrition Environment Measures Survey in Restaurants Spanish Mediterranean Context) is significant, as it increases the number of instruments adapted for the Spanish context to three. Similar to this adaptation, the validity and reliability data for the NEMS-S-MED (Nutrition Environment Measures Survey in Food stores Spanish Mediterranean context) (18) and NEMS-P-MED (Perceived Nutrition Environment Measures Survey to the Mediterranean Spanish Context) (19) questionnaires are high, thus demonstrating their feasibility, validity and reliability.

Future research should use reliable and valid instruments, such as the adapted NEMS-R-MED, in conjunction with the NEMS-S-MED and NEMS-P-MED, in order to collect and compare data. This would contextualize the food environment by combining direct observation and population perception. This approach would support the development and evaluation of future public health policies and interventions related to food access and the promotion of healthy eating. These policies would help to reduce health inequalities, as well as the prevalence of overweight and obesity and non-communicable diseases in the population.

## 5 Conclusions

The adapted NEMS-R-MED instrument is a valid and reliable audit tool for assessing the food environment in restaurants in Spanish Mediterranean contexts. Furthermore, NEMS-R-MED was able to discriminate between restaurant types and neighborhoods with different socioeconomic statuses. Well-designed environmental measurement tools facilitate a better understanding of the environment and support the development of effective policy interventions aimed at increasing both access to and availability of healthy foods.

## Data Availability

The raw data supporting the conclusions of this article will be made available by the authors, without undue reservation.
