# Supplementary material for: Adaptation and evaluation of the Nutrition Environment Measures Survey in Restaurants for the Spanish Mediterranean context (NEMS-R-MED)
Source: Front Public Health. 2026 Jan 26;13:1691374. doi: 10.3389/fpubh.2025.1691374 (PMC12883953; doi:10.3389/fpubh.2025.1691374)
Supplement: Supplementary file 1 [file Data_Sheet_1.docx]

***Supplementary Material***

**Table S1.** Categories and items related with availability of food and food promotion included in the Nutrition Environment Measures Survey in Restaurants (NEMS-R).

| Questions | Number of items | Items included |
| --- | --- | --- |
| Site visit (Observation) | 7 | Restaurant has a salad bar  Signage/Promotions:  - Is nutrition information posted near point-of-purchase, or available in a brochure?  -Do signs/table tents/displays highlight healthy menu options?  -Do signs/table tents/displays encourage healthy eating?  -Do signs/table tents/displays encourage unhealthy eating?  - Do signs/table tents/displays encourage overeating (e.g., all-you-can-eat, super-size, jumbo, grande, supreme, king size, feast descriptors on menu or signage)?  - Does this restaurant have a low-carb promotion? |
| Menú review | 15 | Chips  Baked chips  White Bread  100% wheat or whole grain bread  100% juice  1% Low-fat, skim, or non-fat milk  Total main dishes/entrees  Healthy main dishes/entrees  Total main salads  Healthy main salads  Low-fat or fat free salad dressings main salads  Fruit (w/out added sugar)  Non-fried vegetables (w/out added sauce)  Diet soda  Other healthy or low calorie beverage? |
| Facilitators | 5 | Nutrition information  Healthy entrees identified on the menu  Reduced-size portions  Comments encouraging healthy eating  Other |
| Barriers | 4 | Large portion encouraged  Menu notations that discourage special requests (e.g., No substitutions or charge for substitutions)  All-you-can-eat or "unlimited trips"  Other |
| Pricing | 5 | Sum of individual dishes vs. combined menu  Healthy starters vs. unhealthy  Obligation to order one menu per diner  Reduced or half vs. regular portions  Other |
| Kid’s menu | 13 | Age limit  Healthy options  100% fruit juice  1% low-fat, skim or non-fat milk  Refills on unhealthy drinks.  Healthy garnish available.  Replacing a healthy option with an unhealthy one (e.g., salad for French fries)  Dishes with assigned healthy garnish (e.g., vegetables or salads)  Unhealthy dessert (e.g. sweets, cakes, pies, ice cream, etc.)  Healthy dessert (e.g. fruit)  Nutrition information  Promotion of unhealthy foods  Promotion of healthy foods |

**SUPPLEMENTARY DATA S1. NEMS-R-MED**

**Instrumento de recogida de datos sobre el entorno alimentario en restaurantes**

FECHA DE RECOGIDA: HORA: ID EVALUADOR:

NOMBRE O ID RESTAURANTE:

1. **INFORMACIÓN GENERAL**

1. TIPO DE RESTAURANTE:

1. BAR-CAFETERÍA
2. RESTAURANTE
3. COMIDA RÁPIDA
4. OTROS

EXCLUIDOS: TIPO “TAKE AWAY” O PARA LLEVAR, EJEMPLO: POLLERÍAS, …

1. Fuentes de recogida de la información
2. Visita/observación (si/no)
3. Carta para llevar (si /no)
4. Internet (si/ no)
5. Entrevista (si/no)
6. Tipo de recogida de la información sobre alimentos:
   1. Carta
   2. Menú
   3. Ambos
7. Horarios de apertura:

|  | - LUNES | - MARTES | - MIÉRCOLES | - JUEVES | - VIERNES | - SÁBADO | - DOMINGO |
| --- | --- | --- | --- | --- | --- | --- | --- |
| - 6.00-11.00 |  |  |  |  |  |  |  |
| - 13.00-17.00 |  |  |  |  |  |  |  |
| - 20.00 a cierre |  |  |  |  |  |  |  |

Otro:_________

1. Acceso restaurante: (responder si se tiene la información)

Acceso adaptado (si / no)

Aparcamiento (si/no)

Capacidad sala:_____________

Número de mesas:________

Comentarios:____________

1. **INFORMACIÓN DE CARTA O MENÚ**

En la carta/menú aparecen las siguientes opciones:

6A. Patatas fritas

1. Si
2. No

Comentario:_________________________

6B. Patatas asadas

1. Sí
2. No

Comentarios:____________________________

7A. Pan blanco

1. Sí
2. No

Comentarios:____________________________

7B. Pan integral

1. Sí
2. No

Comentarios:____________________________

8A. Ensaladas saludables (solo vegetales)

1. Sí
2. No

Comentarios:____________________________

8B. Ensaladas con salsas (tipo mayonesa, salsa rosa, salsa césar, etc)

1. Sí
2. No

Comentarios:____________________________

**9. Entrantes**

1. Sí
2. No

9A. Número total de entrantes___________

9B. Opciones saludables (Se entiende como opciones saludables aquellos platos que contengan vegetales, carnes, pescados, huevos, legumbres, cereales integrales… cocinados con preparaciones culinarias que no requieran adición de gran cantidad de grasa (cremas, manteca, mantequilla, margarina, o una gran cantidad de aceite), fritos o rebozados, y en las que se utilicen otras técnicas como vapor, horno, plancha, salteado, etc.)

1. Sí
2. No

9C. Número de entrantes saludables:_________________________

Tipos de entrantes

9D. Ensaladas como plato único

1. Sí
2. No

Número de opciones____________

9E. Verduras sin freír

1. Sí
2. No

Número de opciones____________

9F. Pescado sin freír

1. Sí
2. No

Número de opciones____________

9G. Carne

1. Sí
2. No

Número de opciones____________

9H. Legumbres

1. Sí
2. No

Número de opciones____________

9I. Productos fritos o rebozados

1. Sí
2. No

Número de opciones____________

8J. Otros:_____________

**10. Platos principales**

- - 1. Sí
    2. No

10A. Número total de platos principales___________

10B. Opciones saludables (Se entiende como opciones saludables aquellos platos que contengan vegetales, carnes, pescados, huevos, legumbres, cereales integrales… cocinados con preparaciones culinarias que no requieran adición de gran cantidad de grasa (cremas, manteca, mantequilla, margarina, o una gran cantidad de aceite), fritos o rebozados, y en las que se utilicen otras técnicas como vapor, horno, plancha, salteado, etc.)

1. Sí
2. No

Número de opciones____________

**Tipos de platos principales**

10C. Ensaladas como plato único

1. Sí
2. No

Número de opciones____________

10D. Verduras sin freír ni rebozar

1. Sí
2. No

Número de opciones____________

10E. Pescado plancha

1. Sí
2. No

Número de opciones____________

10F. Carnes

1. Sí
2. No

Número de opciones____________

10G. Legumbres

1. Sí
2. No

Número de opciones____________

10H. Arroces

1. Sí
2. No

Número de opciones____________

10I. Pasta

1. Sí
2. No

Número de opciones____________

10J. Guisos o sopas

1. Sí
2. No

Número de opciones____________

**11. Postre**

a) sí

b) no

Número de opciones____________

11A. Dulce (pasteles, tartas, brownies,…)

Sí

No

Número de opciones____________

11B. Lácteos azucarados (por ejemplo: helados, yogures azucarados, natillas,…)

Sí

No

Número de opciones____________

11C. Lácteos sin azucarar

Sí

No

Número de opciones____________

11D. Fruta

Sí

No

Número de opciones____________

**12. Bebidas**

12A. Zumo de fruta 100%

1. Sí
2. No

Comentarios:____________________________

12B. Néctar/Zumo azucarado

1. Sí
2. No

Comentarios:____________________________

12C. Refrescos azucarados

1. Sí
2. No

Comentarios:____________________________

12D. Refrescos light/zero

1. Sí
2. No

Comentarios:____________________________

12E. Alcohol (vino, cerveza)

1. Sí
2. No

Comentarios:____________________________

12F. Agua

1. Sí
2. No

Comentarios:____________________________

12G. Posibilidad de pedir agua de forma gratuita

1. Sí
2. No

Comentarios:____________________________

12H. Otras bebida saludables no azucaradas (té, infusiones, café,…)

1. Sí
2. No

Comentarios:____________________________

1. **Elementos facilitadores para una alimentación saludable:**

13.A. Información nutricional en la carta

1. Sí
2. No

Comentarios:____________________________

13B. Entrantes saludables fácilmente identificados (con alguna señal, color o iconografía diferente)

1. Sí
2. No

Comentarios:____________________________

13C. Raciones de tamaño reducido o medias raciones

1. Sí
2. No

Comentarios:____________________________

13D. Comentarios que animen a pedir opciones saludables

1. Sí
2. No

Comentarios:____________________________

13E. Otros:___________________

(por ejemplo: disponen y ofrecen envases para llevar la comida no consumida)

1. **Barreras para una alimentación saludable:**

14A. La carta o los letreros del restaurante promocionan las opciones no saludables (aquellos alimentos que suelen considerarse altos en azúcar, sal, grasas y calorías, tales como los dulces, aperitivos de patata, fritos, refrescos, bollería industrial, postres dulces, etc)

1. Sí
2. No

Comentarios:____________________________

14B. ¿La carta o los letreros del restaurante animan a comer más de la cuenta? (por ejemplo: buffet libre, tamaño extragrande en raciones, comidas muy copiosas, o “sin límite de raciones/bebidas”)

1. Sí
2. No

Comentarios:____________________________

14C. Se anima a consumir raciones extragrandes

1. Sí
2. No

Comentarios:____________________________

14D. Posibilidad de aumentar el tamaño de las raciones (con pago extra)

1. Sí
2. No

Comentarios:____________________________

14E. ¿Hay comentarios que disuadan del cambio de ingredientes? Por ejemplo: sin posibilidad de cambio de ingredientes o cambio con cargo adicional

1. Sí
2. No

Comentarios:____________________________

14F. Otros:__________

1. **Comparativa de precios**

15A. Suma de platos individuales frente a menú combinado

1. Más
2. Igual
3. Menos
4. N/A

Comentario:_______________

15B. Precio de entrantes saludables frente a otros entrantes

1. Más
2. Igual
3. Menos
4. N/A

Comentario:_______________

15C. Obligación de pedir un menú por comensal (no se permite compartir menú)

1. Sí
2. No
3. N/A

Comentario:_______________

15D. Precio de las raciones reducidas o medias raciones frente a raciones normales

1. Más
2. Igual
3. Menos
4. N/A

Comentario:_______________

15 E. Precio de las bebidas carbonatadas vs agua

1. Más
2. Igual
3. Menos
4. N/A

Comentario:_______________

15E. Otros:_______________

1. **Menú infantil: Si / no**

Si el establecimiento tiene Menú Infantil, responde las siguientes preguntas:

|  | Marca con una X la respuesta que corresponda | | |  |
| --- | --- | --- | --- | --- |
|  | **Sí** | **No** | **N/A** | **Comentarios** |
| **16A. ¿Hay opciones saludables?** |  |  |  |  |
| **16B. Zumo de fruta 100%** |  |  |  |  |
| **16C. Zumo/Néctar azucarado** |  |  |  |  |
| **16D. Agua** |  |  |  |  |
| **16E. Refrescos azucarados** |  |  |  |  |
| **16F. Refrescos light/zero** |  |  |  |  |
| **16G. ¿Hay recargas gratuitas en las bebidas no saludables?** |  |  |  |  |
| **16H. ¿Hay alguna guarnición saludable**  **(asignados o a elegir)?** |  |  |  | Indicar cuántas: |
| **16I. ¿Puede sustituir una opción saludable por otra no saludable? (ejemplo ensalada por patatas fritas)** |  |  |  |  |
| **16J. ¿Los platos que tienen guarniciones asignadas incluyen un acompañamiento saludable asignado? (ej. Verduras o ensaladas)** |  |  |  |  |
| **16K. Postre no saludable (dulce, pasteles, tartas, helados,…)** |  |  |  | Indicar cuántas opciones |
| **16L. Postre saludable (ej. Fruta)** |  |  |  | Indicar cuántas opciones |
| **16M. Información nutricional (ej: kcal, grasas,…)** |  |  |  |  |
| **16N. ¿Otra promoción de alimentos no saludables?** |  |  |  |  |
| **16Ñ. ¿Otra promoción de alimentos saludables?** |  |  |  |  |

**TABLE S2. NEMS-R-MED SCORE**

Rango total: sin menú infantil: -5 a 23

Rango total completo (con menú infantil): -10 a 31

| **Pregunta** | **Variable** | | **Valor puntuación** |
| --- | --- | --- | --- |
| **Disponibilidad de opciones saludables** | | | |
| 6B | Patatas asadas | | Sí = 1 pt |
| 7D | Pan integral | | Sí = 1 pt |
| 12A | Zumo 100% natural | | Sí = 1 pt |
| 12F y 12H | Agua y otras bebidas no azucaradas (té, infusiones, café,…) | | Sí para cualquiera de los dos = 1 pt |
| 9C | Entrantes saludables | | 1 opción= 1 pt  2-4 opciones=2 pt  +5 opciones=3 pt |
| 9D/10C | Ensaladas como plato único (tanto en entrantes como en plato principal) | | 1 opción= 1 pt  2-4 opciones=2 pt  +5 opciones=3 pt |
| 8A | Ensaladas saludables | | 1 opción= 1 pt  2-4 opciones=2 pt  +5 opciones=3 pt |
| 9H y 10G | Legumbres (tanto en entrantes como en plato principal) | | Sí = 1 pt |
| 10D | Vegetales (no fritos) | | Sí = 1 pt |
| 11C | Lácteos sin azucarar | | Sí = 1 pt |
| 11D | Fruta | | Sí = 1 pt |
|  | RANGO | | 0 a 17 |
| **Facilitadores para llevar una alimentación saludable** | | | |
| 9B y 10B | | Identificadas opciones saludables tanto en entrantes como en plato principal | Sí = 1 |
| 13A | Información nutricional en la carta/menú | | Sí = 1 |
| 13B | Entrantes saludables identificados | | Sí = 1 |
| 13C | Raciones de tamaño reducido o medias raciones | | Sí = 1 |
| 13D | Comentarios que animen a pedir opciones saludables | | Sí = 1 |
| 15B | Entrantes saludables más baratos que entrantes normales | | Sí = 1 |
|  | RANGO | | 0 a 6 |
| **Barreras para llevar una alimentación saludable** | | | |
| 14A | La carta o los letreros del restaurante promocionan las opciones no saludables | | Sí = -1 |
| 14B | La carta o los letreros del restaurante animan a comer más de la cuenta | | Sí = -1 |
| 14C | Se anima a consumir raciones extragrandes/ Posibilidad de aumentar el tamaño de las raciones | | Sí = -1 |
| 14E | ¿Hay comentarios que disuadan del cambio de ingredientes? Por ejemplo: sin cambio de ingredientes o cambio con cargo adicional | | Sí = -1 |
| 15B | Entrantes saludables más caros que entrantes normales | | Sí = -1 |
|  | RANGO | | -5 a 0 |
| **Menú infantil** | | | |
| 16A | ¿Hay opciones saludables? | | Sí = 1 |
| 16B. | Zumo de fruta 100% | | Sí = 1 |
| 16D. | Agua | | Sí = 1 |
| 16 C y 16E. | Bebidas azucaradas (refrescos azucarados y zumos azucarados) | | Sí a cualquiera de los dos = -1 |
| 16G. | ¿Hay recargas gratuitas en las bebidas no saludables? | | Sí = -1 |
| 16H. | ¿Hay alguna guarnición saludable? | | Sí = 1 |
| 16I. | ¿Puede sustituir una opción saludable por otra no saludable? | | Sí = -1 |
| 16J. | ¿Los platos que tienen guarniciones asignadas incluyen un acompañamiento saludable asignado? (ej. Verduras o ensaladas) | | Sí = 1 |
| 16K. | Postre no saludable (dulce, pasteles, tartas, helados,…) | | Sí = -1 |
| 16L. | Postre saludable (ej. Fruta) | | Sí = 1 |
| 16M. | Información nutricional (ej: kcal, grasas,…) | | Sí = 1 |
| 16N. | ¿Otra promoción de alimentos no saludables? | | Sí = -1 |
| 16Ñ. | ¿Otra promoción de alimentos saludables? | | Sí = 1 |
|  | RANGO | | -5 a 8 |
